# Sustainable Transportation and Health

**DOI:** 10.3390/ijerph15030542

**Published:** 2018-03-18

**Authors:** Norbert Mundorf, Colleen A. Redding, Songtao Bao

**Affiliations:** 1 Communication Studies, University Transportation Center, University of Rhode Island, Kingston, RI 02881, USA; 2 Cancer Prevention Research Center, Psychology Department, College of Health Sciences, University of Rhode Island, Kingston, RI 02881, USA; 3 Department of English, School of Foreign Languages, Shanghai Polytechnic University, Shanghai 201209, China; stbao@sspu.edu.cn

We are experiencing a shift in thinking about Transportation and Mobility, which makes this Special Issue on Sustainable Transportation and Health in the *International Journal of Environmental Research and Public Health* especially timely. Mobility is a key factor in both our global economy and our local cultures; it also presents major challenges. The importance of sustainable transport is reflected both in its connections to many, if not most, of the United Nations Sustainable Development Goals, as well as its recognition as a key climate stabilization wedge [1,2,3]. While transport allows us to interact with others near and far, its important upstream and downstream effects on environments, lifestyle behaviors, and individual and population health are increasingly recognized. In particular, single-occupancy vehicle (SOV) transportation has been the norm for decades in developed countries, and it is becoming more prevalent in many emerging economies. For too many of us, driving a gasoline or diesel-fueled automobile by ourselves is the norm. We perceive it as convenient and fast, and often as the only reasonable option. On the other hand, SOV transportation increases pollution, congestion, injury, and death, while reducing physical activity; it also contributes to automobile-centered urban and suburban development. Reduced physical activity, alone, has multiple negative health impacts. Finally, automobile traffic is a major contributor to greenhouse gas emissions and climate change.

This current issue is devoted to research that can contribute to better understanding and promotion of sustainable transportation. Sustainable transportation (ST) includes many diverse mobility modes other than SOV transportation. ST is pivotal for transportation planning, but it also has become a key factor in public health: transportation mode choice affects environmental quality by reducing reliance on automobile transportation and, thus, impacting air quality, neighborhood design, and community life. Importantly, most sustainable transportation modes include physical activity, so important in light of the many public health crises resulting, in large part, from wide-spread inertia. Increased physical activity can improve rates of obesity, diabetes, heart disease, some cancers, and some cognitive and mental health concerns.

Promoting ST alternatives is an important objective not only for transportation planners, but also for public health researchers and policy-makers. Population health includes preventing disease, prolonging life, promoting health equity, as well as physical, mental, and social well-being. Sustainable transportation can improve population health, through individual transportation choices (e.g., using active modes instead of a car), activity patterns (e.g., promoting social participation), neighborhood walkability, and increased exposure to green spaces. Policies that promote sustainable transportation can also impact population choices. Research on these topics can offer important evidence to guide transportation planners, policy developers, community officials, and public health experts.

This Special Issue contains a series of articles exploring diverse aspects of sustainable, alternative transportation modes and their impacts on various aspects of individual and population health. The range of topics comprises human factors, environmental challenges, structural and policy measures which affect the acceptance, functioning, and impacts of sustainable transportation. These papers demonstrated a range of research methodologies as well, ranging from reviews [4,5] and pilot studies [6], to cross-sectional integrative analyses [7] and some randomized trial baseline results [8].

Environmental influences play a key role in the impact of different transportation modes. Authors in this issue explore issues as varied as vibrations from rail [9], nocturnal road traffic noise [10], and ultrafine particle respiration [11]. Reduction of auto emissions is a key ST goal, affecting both local pollutants and greenhouse gas emissions. These articles help us understand such environmental factors better and might inform policy and business decisions.

Structural alternatives are important elements that can encourage or nudge populations to embrace walking, biking, and other modes: complete streets [12], cycle tracks and bicycle parking [13], and urban greenways [14]. A related article explores attitudes towards car-sharing, which can reduce the level of car ownership and is likely to promote alternative transportation choices [15]. The article *Distance, Duration and Velocity in Cycle Commuting* analyzes biking behavior, which may help both planning and promotion of biking (including e-bikes) [16], while another paper examines biking as a means to increase access to jobs and reduce crime [17].

Human factors include attitudes, knowledge, confidence, and readiness to embrace transportation alternatives. Several theoretical approaches to ST were explored here, including the transtheoretical model, the norm activation model, and the theory of planned behavior, among others [4,6,7,18]. These articles provide valuable insights into the many human factors that influence mobility choices. In particular, the transtheoretical model is seen as a promising approach to reach broad populations, although more attention to processes of change and better research methods were recommended [4,6]. Some of these articles integrate theoretical constructs in interesting and innovative ways to predict sustainable transportation behaviors. Although these results are not definitive in and of themselves and are in need of longitudinal replication, they may point towards future transportation research and future theoretical models which are better integrated.

The research included in this issue is from around the globe, including colleagues from Europe [5,8,9,10,11,12,16,18], Asia [7,13,14,15], and the Americas [6,17]. Comparisons across different cultures and environments can provide important information that can inform research and policy choices. For instance, bike commuting is more prevalent in Western Europe and Scandinavia, as well as China, while biking tends to be recreational in the U.S. The realization of the beneficial impacts of active commuting on health and quality of life has led to increased investments in bike facilities, walkability features, car- and bike-sharing options, and public transit/multimodal assets. Cultural factors likely play a key role and are in need of more research replicating and extending the findings presented here.

While the relationship between human activities and nature has been an area of emphasis among western scholars for some time, sustainability has also gained increasing attention from some emerging economies, such as Mexico [17] and China [7,13,14,15]. In particular, the four papers from China reflect its changing transportation landscape and an emerging interest in sustainable transportation and health. These articles address attitudes and behaviors related to car-transport reduction, bicycle tracks and parking, urban greenway use, and free-floating car sharing mode from different perspectives. China is facing serious health problems related to transportation. In 2013 road traffic mortality rate (per 100,000 population) in the U.S. was 10.6, while it was 18.8 in China, even higher than the global average of 17.4. In 2012 the mortality rate attributed to household and ambient air pollution (per 100,000 population) was only 12.1 in the U.S., while in China this number reached an astounding 161.1, more than 1.7 times higher than the global average of 92.4 [19]. Fortunately, the Chinese government has realized the high significance and urgency of tackling these issues for a “Healthy China”. At the National Health Conference in August 2016, President Xi stressed that health is a prerequisite for all-round development, a precondition for economic and social development and a common aspiration of all people [20]. Health became an explicit national political priority with the approval of the Healthy China 2030 Planning Outline, the first national medium-to-long-term strategic health plan since 1949 [21]. Finally, this also indicates the political commitment of China to participate in Global Health Governance, and meet the United Nations Sustainable Development Goals agenda [2].

Future research may incorporate additional considerations, such as cultural differences and societal factors that impact sustainable and health behaviors, stressing the links between transportation and health issues and societal trends, such as decreasing crime, improving economic development, and increasing equity and social justice. Additionally, adaptations of various research methodologies may be required to reflect vastly different communication environments and research practices in developing countries and emerging economies.

The focus of the studies in this issue was mainly on individuals; future research on organizational, governmental, and institutional levels could help to ensure a greater emphasis on prevention rather than treatment. Although individual behavior change is essential, organizational and policy changes are also important factors to include in facilitating individual and population level changes. We hope that this Special Issue will encourage future research in various aspects of sustainable transportation and health and lead to insights, which, in turn, provide guidance to politicians, planners, transportation, and public health professionals to help reach our goals of healthy populations in a healthy environment.
